# Spatial localisation and sensing in two dimensions via metasurfaces

**DOI:** 10.1038/s41598-024-75218-2

**Published:** 2024-10-15

**Authors:** Georgiana Dima, Christopher John Stevens

**Affiliations:** Department of Engineering Science, University of Oxford, Parks Road, Oxford, OX1 3PJ UK

**Keywords:** Electrical and electronic engineering, Characterization and analytical techniques, Imaging techniques

## Abstract

In this study, we introduce a two-dimensional metasurface sensor designed to detect, locate and distinguish between different objects placed in its near field. When an object is placed on the metasurface, local changes can be detected in one or more of the structure’s meta-atoms. This interaction generally modifies the inductance of the meta-atom, resulting in changes to the overall input impedance of the surface. We derive the properties of the structure and its behaviour in terms of superposition and demonstrate that observing the meta-surface from a single point is sufficient for unambiguous localisation and identification. To model these changes effectively and identify the position of an object, we employ a neural network machine learning algorithm. Our approach enables accurate localisation of all studied objects, with a precision exceeding $98\%$. Additionally, the distinct signatures of the objects allow for separation between them with an accuracy of over $97\%$. The potential applications of this platform extend to foreign object detection on metasurfaces for wireless power transfer, providing proximity detection for many surfaces such as clothing, car bodies and robotic carapaces. Furthermore, our research suggests the feasibility of implementing a touchscreen type interface requiring only a single waveguide connection.

## Introduction

Metasurfaces have long been known to support a variety of surface waves that act to distribute signals widely across them. Being formed from arrays of identical meta-atoms, such surfaces derive all of their properties from these meta-atoms and their interactions. The foundation of this research field was established at the beginning of the $21^\textrm{st}$ century through the work of Smith et.al.^1^, who combined rods, which were proved to exhibit negative permittivity by Rotman^2^ and Pendry^3^, with split ring resonators, proven by Thompson^4^ and Pendry^5^. Our particular interest is in meta-atoms constructed using discrete circuits, which exhibit electrical resonances. These surface’s waves have been described in detail in^6^ and exploited as channels for wireless power transfer^7,8^ and data transfer^9^ amongst others. One application that has gained popularity in recent years is sensing techniques which include displacement sensors^10–12^, conductivity, permeability and permittivity sensors^13–16^, as well as voids and cracks identification^17,18^. In this article, we focus on a two-dimensional metasurface sensor that is capable of object localisation and characterisation.

In this work, we are particularly interested in open metasurfaces where the interacting force fields of the meta-atoms surround them, meaning that their electromagnetic fields are exposed to the nearby environment. This, in turn, means that nearby objects can greatly influence the behaviour of the individual meta-atoms. In previous work, we have exploited this property to create a one-dimensional sensor based on the perturbation of a linear arrangement of magnetic meta-atoms^19^. Yan et al.^19^ present an algorithm that can estimate the position of an object with high conductivity based on the changes in the standing wave pattern. This sensing area was explored further in literature, where a one-dimensional array was used for sensing the position of a defect using time-domain reflectometry^20–22^.

Similarly to^19^, the technique discussed in this article relies on changes in self-inductance (*L*) and resistance (*R*) of a single resonant element when an object is placed in its vicinity. As a result, additional reflections occur from the affected meta-atom, which alter the standing wave pattern. With an asymmetrical excitation position, the reflections from each meta-atom are unique. The choice of excitation positioning will be further explored in the Methods section. In^19^, the number and location of peaks in a one-dimensional array were used to determine the location of an object. This human-accessible algorithm was unable to localise objects placed 4.5 mm above a one-dimensional array. This suggests that a more complex approach is necessary to bypass the intrinsic limitations of the problem and generalise magneto-inductive localisation for a two-dimensional array. Here, a machine learning algorithm in the form of a neural network with one hidden layer is explored as a viable option for modelling the complex changes in the standing wave pattern occurring in such a system. Machine learning methods have gained popularity recently when designing and optimising metasurfaces^23–28^ or metasurface biosensors^29,30^.

The element of novelty in this work is the ability to exploit the well-established concepts of magnetoinductive waves and scattering to develop a two-dimensional localiser, which can map frequency domain variations onto the spatial domain. An additional element of novelty is the use of a machine learning algorithm to model the changes in the magnetoinductive wave spectrum when objects interact with the array. The use of such a powerful modelling tool enables the usage of a single measurement point, which results in exciting opportunities such as more sensitive and more measurement-efficient touchscreen arrays^31^. This technology is a highly popular non-mechanical human-machine interface that has been developing rapidly over the last two decades and is now widely used, including in most of our day-to-day electronics such as smartphones, smart watches, tablets or even vending machines. As described in^19^, touchscreen arrays usually consist of multiple electrodes arranged in a dense grid. When the user touches the screen, the local mutual capacitance between adjacent electrodes is changed. By interrogating the electrodes in pairs, the user-specified location is determined. This requires a large number of measurements as well as relatively complex electronics^32^. Our magneto-inductive sensing alternative requires only one spectrum measurement, reducing the physical complexity of the system. In the system described, only a single impedance spectrum measurement is required from one waveguide connected to the device at one point in order to locate a touch point on the metasurface.

Another promising application of a two-dimensional localisation sensor is in the detection of foreign objects on metasurfaces. This technology can be utilised for identifying defects on the exterior of stealth aircrafts and spacecrafts, where maintaining the integrity of the surface is crucial for performance and safety. Additionally, it can be applied to wireless charging platforms to detect and locate foreign objects, ensuring efficient and safe operation by preventing interference or damage caused by unintended items.

This work begins by describing the method used for localisation and object separation. First, the analytical principles involved are detailed, followed by the experimental layout and the machine learning algorithm. The next section, ’Results’, presents analytical results for localisation, discussing the impact of different excitations and noise. Experimental localisation and separation results between objects are then presented. The final section discusses the conclusions of this work and future research avenues.

## Methods

### Analytical description

In order to elucidate the mechanism by which our sensor works, we consider a simple two-dimensional metasurface of size $M\times N$ formed from identical meta-atoms (typically resonant circuits coupled together via mutual inductance). As an example, we could consider any resonator that can be modelled as an *RLC* circuit with resistance *R*, inductance *L*, and capacitance *C*, such as loaded multi-turn coils. When excited individually, without the presence of an object, each meta-atom has a resonant frequency, $f_\textrm{res} =1/(2\pi \sqrt{LC})$ and a quality factor, $Q = R/(2\pi f_\textrm{res}L)$. When placed in an array, the meta-atoms couple to one another through their mutual inductance, *M*, which results in a coupling between them described by the dimensionless coupling $\kappa = 2M/L$^6^. This allows for the propagation of two-dimensional magnetoinductive waves, which typically have a very short wavelength. As a result, if one launches a wave into such a planar waveguide at any point, $m_F, n_F$, it will excite standing waves. If the input impedance spectrum is measured at this point on the surface, a series of peaks and troughs will characterise the behaviour of the structure. The input impedance spectrum is defined as the ratio between the excitation voltage spectrum and the current in the excited meta-atom:

$$\begin{aligned} Z_\textrm{in} = \dfrac{V_\textrm{in}}{I_\textrm{in}} \end{aligned}$$

**Fig. 1:**
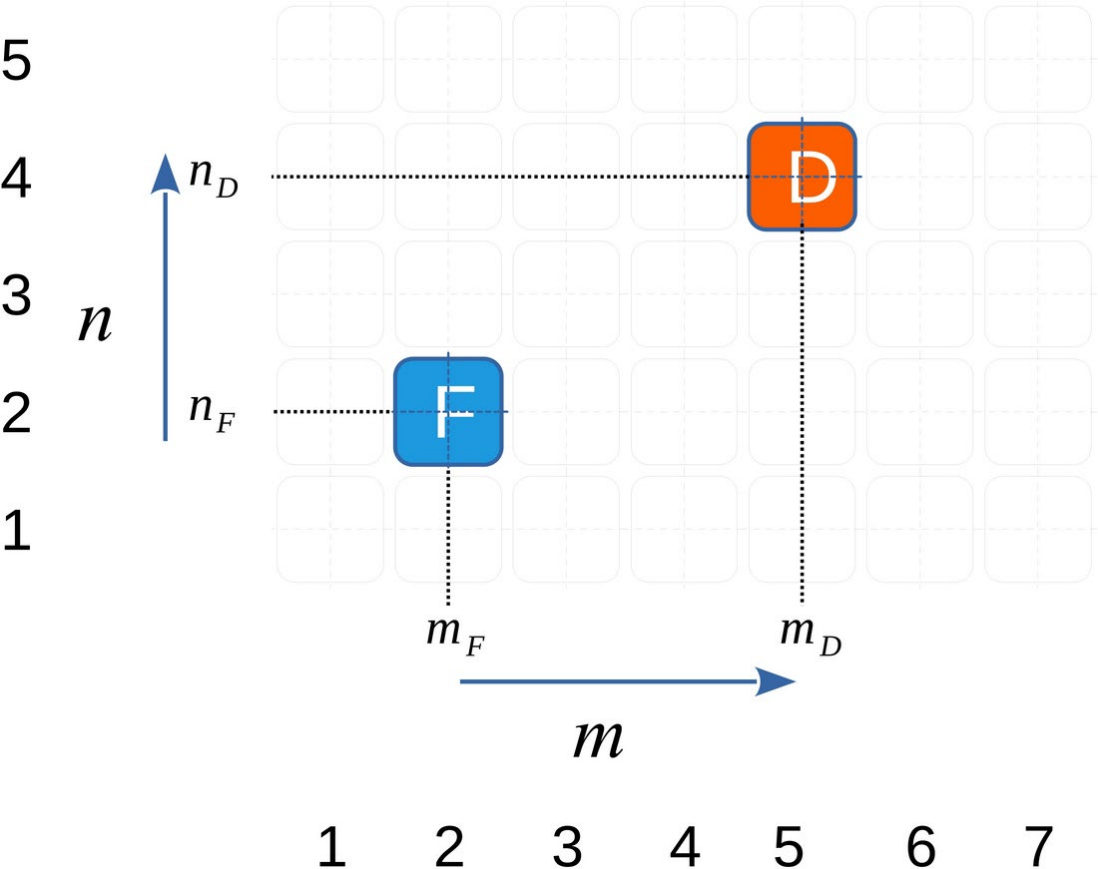
Map of a 2D metasurface with a signal source - Feed ’F’ located at $m_F,n_F$ and a defect ’D’ at $m_D,n_D$.

In this case, we will pick a point on the surface and measure the reflection spectrum in order to probe the whole surface in one step. If an object interacts strongly with one of the meta-atoms in the metasurface, say by changing its inductance, it generates a lattice defect by de-tuning it. In consequence, that meta-atom becomes a reflector and scatters arriving waves, modifying the pattern of the excited standing waves. From a measurement point of view, this results in a change to the input impedance spectrum. At first glance, this seems to require the determination of the point scattering coefficients for each possible type of interaction. In general, this would be a difficult set of calculations to complete and would still require evaluation of the new standing wave patterns for a more complex structure than the initially uniform regular surface. Instead of calculating the scattering properties of point defects, we consider the way in which an object interacts with a meta-atom. For example, we look at the most extreme case where an interaction completely cancels the current that would have circulated in an unperturbed lattice (creating a “vacancy”). Here, the result is that we now have a meta-atom with $I_{m,n}=0$. This suggests that an alternative approach to determining the impact of this perturbation on the feed point is to model the *vacant* meta-atom with a virtual source placed in the otherwise unperturbed cell meta-atom. The extra current generated in the meta-atom is set to be the exact anti-phase of that arriving from the original feed, $N_\textrm{ex}$, at that point in the unperturbed lattice. In other words, instead of a missing or modified meta-atom, we have to model a second source with a particular phase and amplitude being injected at the location of the defect. This is shown in the diagram of Fig. 1. Hence, the current circulating in the feed meta-atom at location $m_F,n_F$ when a defect is present at location $m_D,n_D$ can be expressed as:

1 $$\begin{aligned} I_{m_F,n_F}=\frac{V_{\textrm{source}}}{Z_{in}(m_F,n_F)}+I'_{m_F,n_F}(m_D,n_D)=\frac{V_{\textrm{source}}}{Z'_{in}(m_F,n_F)} \end{aligned}$$

where $Z_{in}(m_F,n_F)$ is the unperturbed metasurface’s input impedance as measured at the feed location, $N_\textrm{ex}$, and $V_{\textrm{source}}$ is the source amplitude being applied at the feed point. The extra current $I'_{m_F,n_F}(m_D,n_D)$ is that current which arrives at the feed point from the virtual source placed at the defect location, launched in antiphase as discussed above. $Z'_{in}(m_F,n_F)$ is the new input impedance that is measured for the perturbed metasurface. The essence of Eq. 1 is that the perturbed metasurface behaves as though an extra current is appearing at the feed in a superposition-like manner. This can be read by measuring not the input reflectance but the input admittance. We can derive this from Eq. 1 by dividing both sides by the source amplitude $V_{\textrm{source}}$:

2 $$\begin{aligned} Y_{in}(m_F,n_F)=Y_0+Y'_{m_F,n_F}(m_D,n_D) \end{aligned}$$

where $$Y_0$$ is the unperturbed input admittance and $$Y'_{m_f,n_f}(m_d,n_d)=I'_{m_F,n_F}(m_D,n_D)/V_{\textrm{source}}$$ is the *referred admittance*. By this, we mean the extra admittance that is measured at the feed meta-atom $$m_F,n_F)$$ when a defect is formed at meta-atom $$(n_D,m_D)$$. If the defect is partial - not a complete cancellation of a perturbed meta-atom’s current but merely a reduction, then the result is the same, but the form of the referred admittance will change. In other words, to determine the current state of the metasurface, one can measure the input admittance, subtract the known unperturbed admittance $$Y_0(m_F,n_F)$$, and then analyse the remaining $$Y'$$. This situation can be generalised to more than one perturbed meta-atom, where:

3 $$\begin{aligned} Y'(m_F,n_F)=a_1 \ Y'_{m_F,n_F}(m_1,n_1)+ a_2 \ Y'_{m_F,n_F}(m_2,n_2)+.... =\sum _p a_p Y'_{m_F,n_F}( {m_{p}},{n_{p}} ) \end{aligned}$$

in which the admittances $Y'_{m_F,n_F}({m_{p}},{n_{p}})$ are those generated by virtual sources placed at the locations $m_{p},n_{p}$ and the complex coefficients $a_p$ are their relative amplitudes. If an object is placed on the metasurface that partially cancels out the current in a particular meta-atom, as may happen for a metal object smaller in size than the meta-atom, then the values of these coefficients will decrease in magnitude. Even for two or more $100\%$ defects, the coefficients may be less than unity with the current from one defect reinforcing that from the other. These coefficients may also be complex, representing the possible phase differences between defects or object-induced changes to their complex impedances.

Despite this complication, the implication of Eq. 3 is that the net input admittance for multiple defects is, in fact, the sum of a series of admittance terms. By subtracting the unperturbed input admittance, the remainder is the sum of a number of scaled referred admittances.

**Fig. 2:**
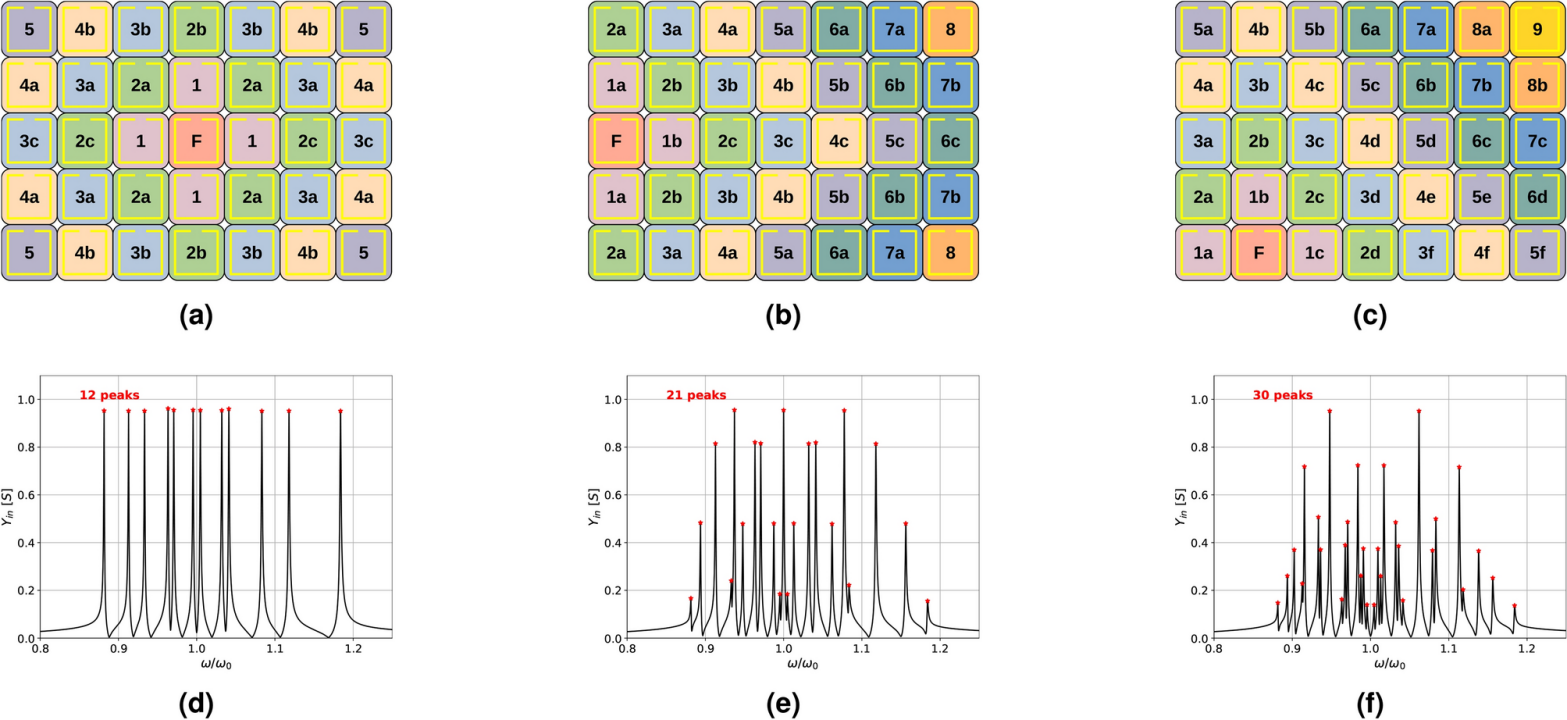
Three identical metasurfaces driven from different feed locations (F). (**a**) Central, (**b**) Edge & (**c**) Asymmetric and their corresponding input admittance spectra (**d**,**e**,**f**). Meta-atoms in each case are labelled according to their Manhattan distance from the feed and their uniqueness in terms of symmetry.

The symmetry of the metasurface also plays a key role in controlling the ability to uniquely identify a perturbation’s location, as shown in Fig. 2. Here, we show the configurations and input admittance for a metasurface formed from resonators with a Q factor of 1000 and a relatively strong first-order coupling of $\kappa =-0.16$. With a structure of high symmetry, like the centrally fed metasurface in Fig. 2a, there are multiple sets of non-unique meta-atoms. At a Manhattan distance (or m-distance)^33^ of 2 steps, for instance, one finds four identical locations on the immediate diagonal from the feed (2a), two on the edge of the metasurface (2b) and two additional ones inside the surface (2c). In these three cases, a defect formed at each of these (2a,2b,2c) would generate a different standing wave pattern despite being identical distances from the feed; however, since there are four equivalent (2a) locations, these will have identical effects on that pattern and hence on the input admittance measured at F. A defect formed at one of the 2a meta-atoms will generate the same modification to the standing waves, whichever of the four it is on. This degeneracy between equivalent locations is a problem for high-symmetry systems. Figure 2a has 12 unique locations, which can also be seen in the 12 peaks present in the admittance spectra of the surface calculated for the central feed point, shown in Fig. 2d. Moving the feed location changes the situation. Figure 2b with an edge feed and 2c with asymmetrical feed are progressively lower symmetry configurations. Now the number of unique locations in the structure increases to 21 (2b & 2e) and then 30 (2c & 2f). This is consistent with the symmetry - the edge-fed 2b structure has a horizontal line of symmetry, whilst the asymmetric feed 2c has none.

These results imply that the number of non-degenerate unique locations that are present in the standing wave pattern is identical to the number of peaks detected in the input admittance spectrum. Fewer degenerate locations (lower symmetry) result in more peaks appearing. On this basis, we would expect the asymmetric case to show 35 peaks, but we are only able to identify 30 because of the first neighbour coupling assumption and because some of the peaks are very close and become difficult to resolve.

Given that we can now construct a non-degenerate standing wave structure, we anticipate that this kind of asymmetric fed structure should provide the possibility to unambiguously locate a defect based on its referred admittance spectrum measured at a fixed feed point. Recording the admittance at one asymmetric feed location should provide the capability for two-dimensional localisation of defects as they are formed by proximity interactions with objects on the metasurface. We can then design the meta-atoms of that surface to be sensitive to a wide variety of conductive, magnetic or dielectric objects.

### Experimental design

**Fig. 3:**
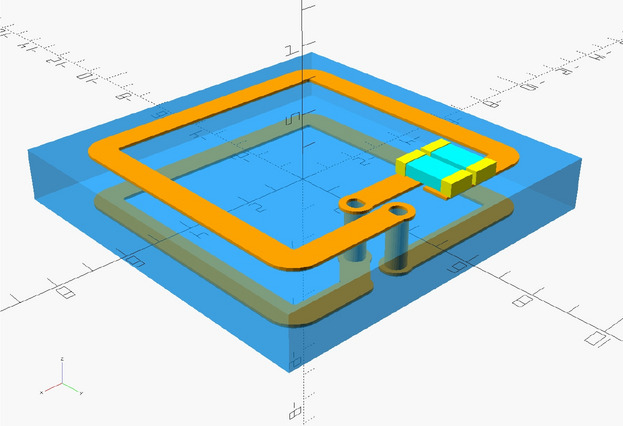
3D model of the meta-atom used in the experimental implementation of the sensing platform.

**Fig. 4:**
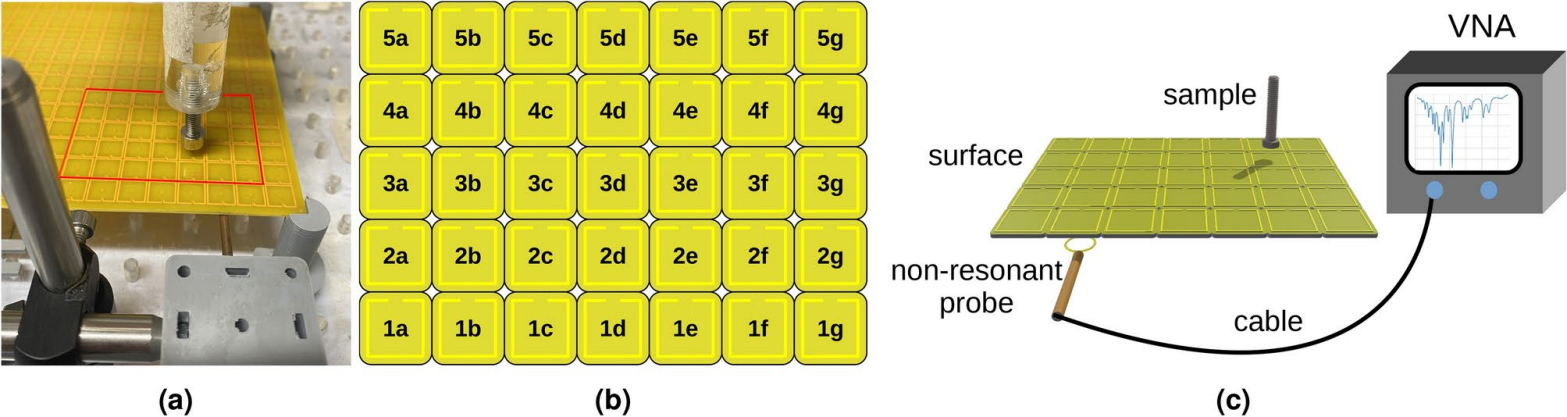
Experimental diagrams: (a) photograph of structure where red lines delimit the tuned meta-atoms from the rest, (b) surface diagram with labels for each meta-atom name, and (c) experiment schematic.

To test the theory, we have designed an experiment following the symmetry of Fig. 2c. A 3D rendering of the meta-atom used is shown in Fig. 3. Each of our meta-atoms is a two-turn, square, helical resonator with a helical pitch of 1.6 mm and an outer square dimension of 10 mm. These are formed using a two-layer printed circuit board with two vertical connections. Each of the helical resonators is tuned with a 10 pF capacitor, from 657 to 183 MHz, whilst raising the Q factor from 44 to 132. The lattice period of the metasurface is 11 mm whilst the width of the tracks is 0.6 mm, their thickness is $35 \ \mu$m, and the gap between adjacent tracks is 0.4 mm. Our approach started with a large array of these meta-atoms, out of which a $7\times 5$ grid was populated with capacitors, as shown in Fig. 4a. The additional via in the centre of each meta-atom is used to mark the centre and is not connected to the resonator. The coupling coefficient between nearest neighbours in our structure was measured to be $\kappa = -0.16$^34^. Rather than using the x-y coordinates of the helical resonators, we instead give each of the 35 resonators a unique label, as shown in Fig. 4b to simplify machine learning implementation.

Figure 4 cshows a cartoon for the experiment. A non-resonant inductive loop was placed below resonator **1b** corresponding to the asymmetric feed location of Fig. 2c and connected via a coaxial cable to one of the ports of a Vector Network Analyser (VNA). A single non-averaged reflectance ($S_{11}$) measurement was collected for each unique object location as that object was moved across the surface. The scanned object was positioned using a simple x-y-z gantry scannner over the centre of each of the 35 tiles with its altitude *h* scanned over a range of $0-10$ mm in steps of 0.25 mm.

The eight objects used in experiments are described in Table 1. Since our metasurface was largely a magnetically coupled array of resonant inductors, we used metallic objects so that they have a strong interaction with the meta-atoms. Different types of metals and sizes were explored in order to test the capability of the detection method. Size-wise, there were two types of objects: large ones (which are comparable in size to the period of the structure studied) and small ones (whose diameter is roughly half that of the period). In addition, a lossy ferrite plate with dimensions comparable to the period was also used.

**Table 1:** Description of objects used experimentally.

Id	Name	Description
1	Large aluminium	Disc $\varnothing = 10.3$ mm, thickness = 1.0 mm
2	Large brass	Disc $\varnothing = 10.3$ mm, thickness = 1.2 mm
3	Large steel	Disc $\varnothing = 10.3$ mm, thickness = 0.8 mm
4	Small aluminium	Disc $\varnothing = 6.8$ mm, thickness = 1.0 mm
5	Small brass	Disc $\varnothing = 6.6$ mm, thickness = 1.2 mm
6	Small steel	Disc $\varnothing = 6.6$ mm, thickness = 0.8 mm
7	Ferrite	Plate $8.4\times 11.5\times 4.7$ mm
8	SS bolt	M6 Hex head Stainless Steel Machine screw

**Fig. 5:**
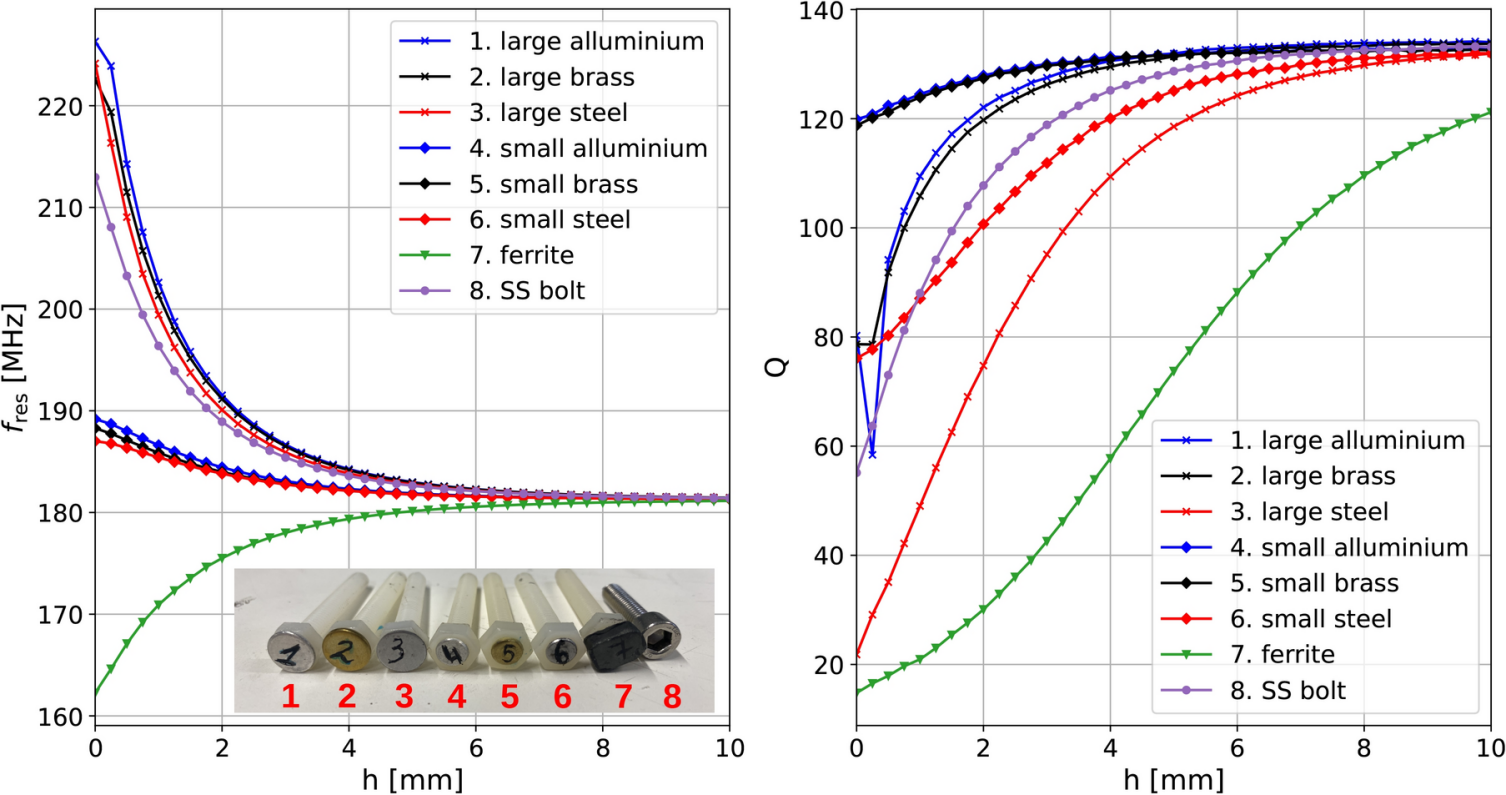
Variation of resonant frequency, $f_\textrm{res}$ (left) and quality factor, *Q* (right) of a tuned double layered spiral resonator with vertical location, *h*, of different objects.

When each of these objects is positioned in proximity to an individual helical resonator, they modify the magnetic field generated by currents circulating in the resonator, resulting in a change to the complex self-inductance. This is evident as a shift in the resonant frequency, $f_\textrm{res}$, and quality factor, *Q*, as described in^35,36^.

The impact of each one of the objects was studied by placing two non-resonant coupling loops (5mm diameter) near a single isolated meta-atom and performing transmission $S_{21}$ measurements between them as the object’s altitude, *h*, above the meta-atom is varied. For accurate measurements, the data collected was averaged eight times and background transmission measurements without the meta-atom but with the object moving were also collected and subtracted from the averaged data to isolate the meta-atom response. The phase method described in^37^ was utilised in order to extract $f_\textrm{res}$ and *Q* for each object and each vertical position. The variation of these parameters is shown in Fig. 5 along with a photograph showing all the objects (inset). Perhaps unsurprisingly, larger objects (metal 1,2,3,8 and ferrite 7) display the most significant change (increase for metals and decrease for ferrite) in resonant frequency, $f_\textrm{res}$, and decrease in quality factor, *Q*. Smaller metallic objects (4,5,6) produce comparatively smaller increases in resonant frequency and smaller decreases in Q factor. Considering resonant frequency changes, the distinguishing factor between metals appears to be their conductivity and size. When comparing objects of similar size, the magnitude of change is arranged based on the object’s conductivity, with aluminium (most conductive) showing the most substantial change and steel (least conductive) the least. Loss dominates the quality factor changes, with conductive objects contributing to some losses, but the impact of permeability is much stronger, with ferrite and mild steel objects exhibiting the most dramatic alterations.

Since the data for these interactions exhibits a good variation between the materials and sizes of objects, it seems reasonable to expect this kind of sensing to allow us to categorize objects effectively, permitting their identification.

### Localisation and identification via machine learning

As described above, accounting for the partial disruption of each helical resonator is a complex analytical problem that is further complicated by experimental and analytical differences stemming from approximations and higher-order coupling. A different, more powerful method of modelling these changes is therefore required in order to show the proof of concept for the two-dimensional localisation. A three-layer neural network was chosen due to its ease of implementation through the PyTorch environment and high efficiency. This method was chosen to show its potential, and further improvements to the architecture could be made for more complicated systems. The two alternative methods considered were logistic regression and gradient-boosted trees. Whilst the complexity of the logistic regression is low, making it an attractive method from an ease of implementation standpoint, the linear decision boundary means that this method is unable to model the non-linear aspects of the system and is, therefore, expected to result in low accuracies. Gradient-boosted trees were a better option, capable of capturing the non-linear effects. However, neural networks were found in the literature to perform inference at a faster speed when compared to similarly complex gradient boosting methods^38–40^. This is an important feature for our system as we aim to create a low-latency prediction model. A convolution neural network (CNN) could also be used and would be appropriate given that this can also be seen as a two-dimensional prediction problem. Given the number of observations, this is an unnecessarily complicated approach.

**Fig. 6:**
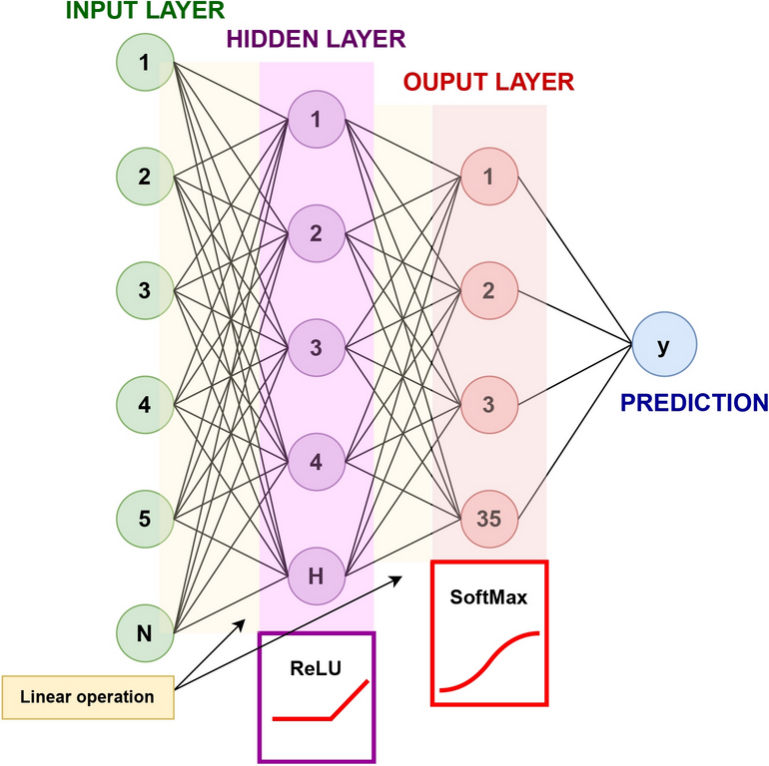
Schematic representation of layers used in a neural network for localisation.

The proposed neural network is trained on a subset of the measurement data and is able to infer information about positions in data that has not been previously presented to it. We used the same neural network approach to evaluate simulated and measured data. Simulated data was derived from circuit modelling where defects are introduced by adjusting a meta-atom’s LRC parameters. Experimental data is recorded using the methods described earlier and shown in Fig. 4c A background measurement of the unloaded array is subtracted from the $S_{11}$ data recorded from the loop coupler (Fig. 4c to ensure higher sensitivity to changes. For each vertical position, one complete scan of the object over the metasurface is completed, and each of these planar scans is one layer of data. The total number of samples in the full dataset is 1435. The data layers are divided between training and testing datasets with a ratio of 1 : 3 between them and labelled according to the desired output, either by location to train a localisation network or by object type to train the identification network. This split was chosen to minimise the amount of data required for training. The training dataset is represented by all the data points at fixed vertical positions ($h = 0, \ 1, \ 2, \dots$ mm) while the test data covers intermediate positions ($h=0.25,\ 0.5,\ 0.75,\ 1.25\dots$).

A PyTorch Neural Network^41^ was used to implement the system. All the $N = 1601$ points in each recorded spectrum become features of the training model. We use a three-layer neural network with input, hidden and output layers. A linear operation, defined as a weighted sum of the inputs followed by the addition of a bias term, is performed on the input layer, resulting in an additional hidden layer (here, of size $H = 100$). A ReLU activation function is applied to the hidden layer values, which introduces non-linearity and allows the network to model more complex data. Our characterisation has shown that this is essential given the strongly non-linear variation shown in Fig. 5. An additional linear operation is applied to the hidden layer, and a SoftMax activation function is used on the output layer. This assigns a probability for each of the existing outputs and allows for the object with the largest probability to be chosen as the prediction. A schematic of the layers is shown in Fig. 6 for the case of position localisation. The Adam optimizer and CrossEntropyLoss function were used, with training data divided into batches of size 25. An initial learning rate of $2 \times 10^{-5}$ was used for $10^4$ iterations, decreasing by 0.9 every $10^4$ iterations to accommodate smaller variations. The learning rate was chosen after several iterative tests to be the optimal one.

## Results

### Analytical localisation

We first considered a noiseless system by using a simulation of the metasurface to test the method. This was achieved by creating an analytical model (using circuit theory^42^ for the magnetoinductive metasurface and using it to assess if the SS-bolt could be located. The impact of the SS bolt was simulated by modifying the resonant frequency and the quality factor of the helical resonator, where the SS bolt is perturbing the surface with values obtained experimentally, as shown in Fig. 5. The data used for training in this case was the magnitude of the difference between the computed input admittance of the loaded surface and the computed input admittance of the unloaded surface, $|\Delta Y_{11}|$.

**Fig. 7:**
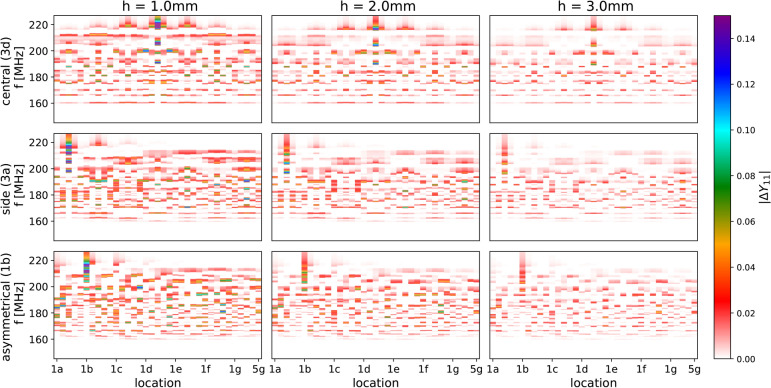
Variation of absolute value of analytical input admittance, $|\Delta Y_{11}|$ with frequency, *f*, and location for different vertical locations ($h = 1, \ 2, \ 3$ mm) and different feed points (central - **3d**, side - **3a** and asymmetrical - **1b**).

Figure 7 shows the variation of $|\Delta Y_{11}|$ with location (labelled according to Fig. 4b and frequency for three different vertical locations ($h = 1, \ 2, \ 3$ mm) and for the three different feed locations as shown earlier in Fig. 2a,b and c. The first noteworthy feature is the difference between the feed locations. When the feed is placed in the centre ( top row $F =$**3d**) or on the side (middle row $F =$**3a**), the spectra for each object location are not unique, as is clear from the symmetry of the maps. This is not the case for the asymmetrical excitation (bottom row $F =$**1b**), where each of the vertical slices is unique. Secondly, increasing the object altitude results in a lower contrast map, but it still has the same symmetry and the same peaks as a function of position. Hence, the pattern of admittance peaks as a function of object location is essentially independent of the strength of the object’s interaction with the surface. The contrast in the pattern is only a function of the object’s altitude. Hence, the pattern can provide location information, and the contrast can be used to obtain object size/classification capabilities.

**Fig. 8:**
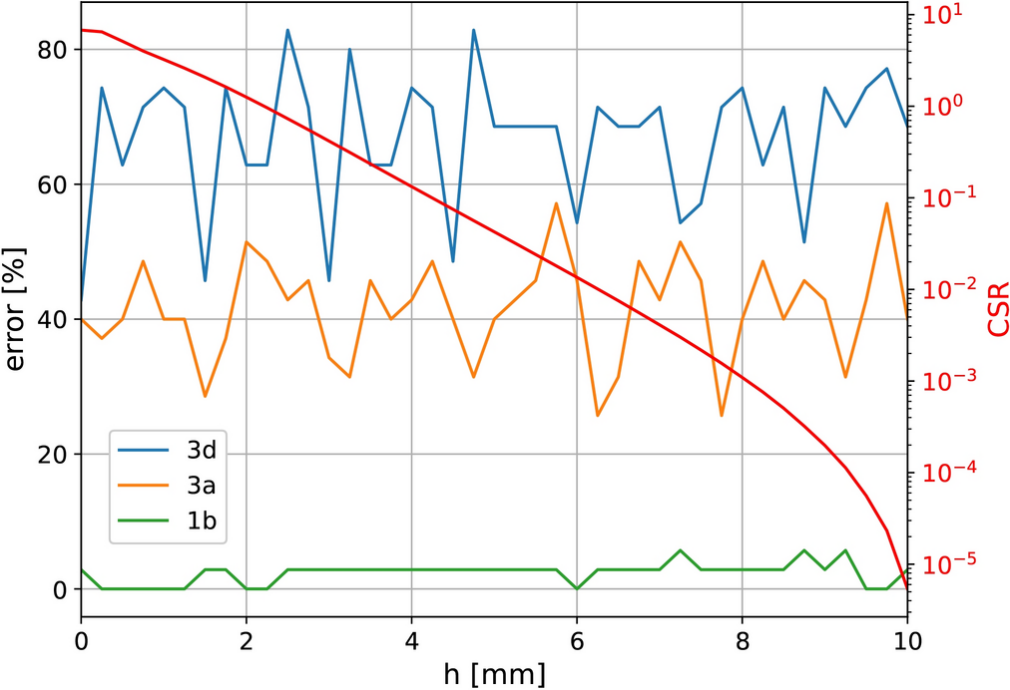
Error in object localisation for three different excitations (3d, 3a and 1b) (left axis) and mean change in the $Y_{11}$ parameter (CSR) as a function of vertical location, *h*.

**Fig. 9:**
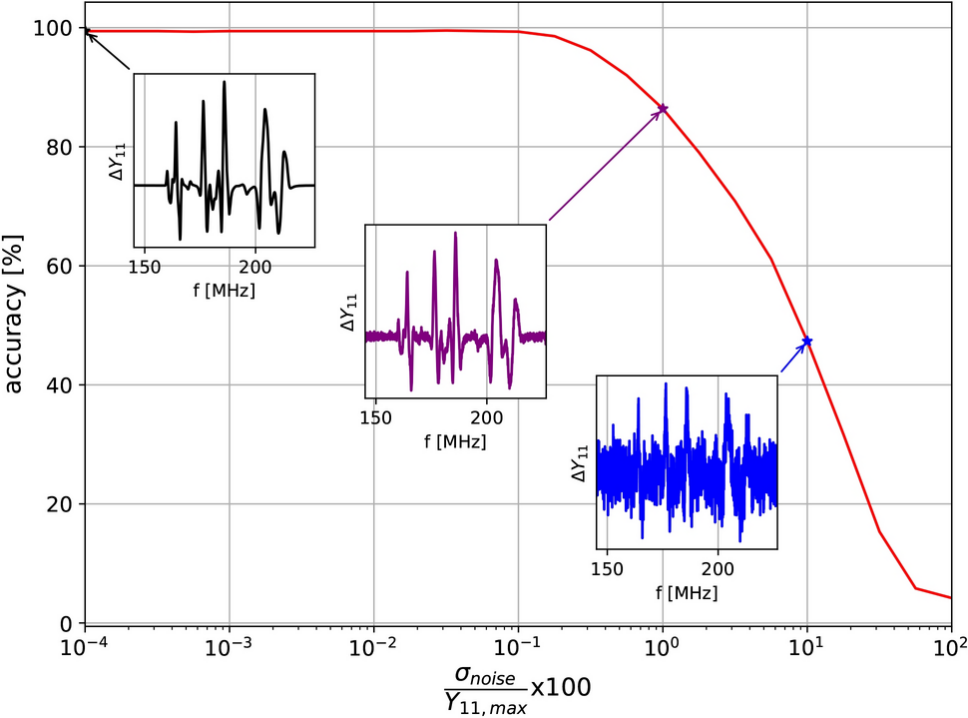
Accuracy in localisation for different ratios between noise standard deviation and maximum unloaded input impedance.

Figure 8 shows the percentage of mislabelled horizontal locations for each vertical position, *h*. The three lines (blue, orange and green) represent the three feed locations. The number of iterations for the training was $10^4$. The additional red line is the mean change to signal ratio (*CSR*) over all positions, which is defined as in Eq. 4, where *i* and *j* are coefficients for the frequency points and location points.

4 $$\begin{aligned} CSR = \dfrac{\sum _i\sum _j{|\Delta Y_{11,i,j}|^2}}{\sum _i\sum _j{|Y_{11,i,j}|^2}} \end{aligned}$$

CSR is a simple way to express the strength of the interaction between an object and the metasurface, in this case, as a function of object altitude *h*. In this case, the strength falls rapidly until $\approx 8$ mm, after which it essentially disappears. For this reason, it is reasonable to use the measurement recorded for $h=10$ mm as a baseline for the unperturbed structure.

The average error in extraction for the full data set when the surface is centrally excited is $66.8\%$, which is in line with the fact that a centrally fed $7\times 5$ structure has only 12 unique locations (i.e. $34.3\%$ unique locations leading to an expected error of at least $65.7\%$). The same is true for the edge excitation, where the error is $41.6\%$ for $60\%$ unique locations. In the asymmetric case, an error of $2.4\%$ is achieved, confirming that with an appropriate feed point, our method can accurately identify locations. The result can be further improved by increasing the number of iterations. In terms of *CSR*, as the altitude, *h*, does not seem to generate a variation trend, one can infer that accurate detection is possible down to a ratio of $10^{-5}$.

Another avenue that we explored using the analytical model was the impact of noise on the data. Additive white Gaussian Noise of mean 0 and standard deviation $\sigma _\textrm{noise}$ was applied to the simulation results. The percentage ratio between the standard deviation of the noise and the maximum value of the unloaded array input admittance spectra with asymmetrical excitation was varied between $10^{-4} \ \textrm{to} \ 100\%$. Figure 9 shows the localisation accuracy variation with this ratio as well as object $|\Delta Y_{11}|$ spectra for three noise values. The localisation accuracy starts to degrade when the ratio is $0.1\%$, which is roughly equivalent to an SNR value of 60 dB. Most VNA measurements rarely fall below 40 dB^43^, which through the same logic would correspond to an accuracy of $86\%$. In this manuscript, the signal-to-noise ratio is roughly 80 dB, which indicates that noise should not impact the results in any significant way.

### Experimental localisation

**Fig. 10:**
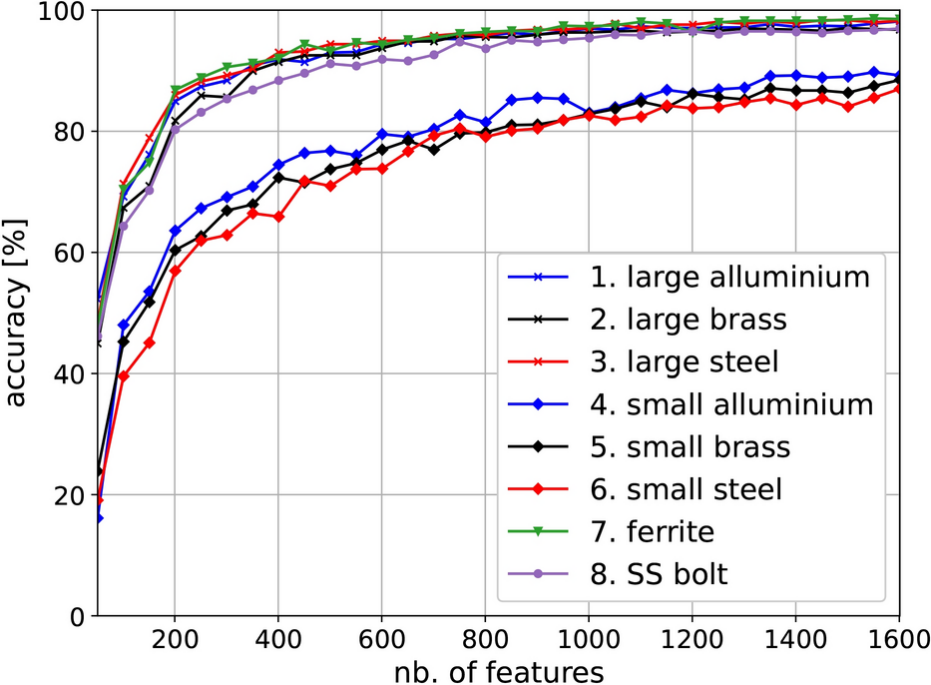
Accuracy variation for all experimental objects with a number of features of the neural network. The number represents an average over all vertical locations.

Having verified the localisation method using noiseless and noisy simulation data, we proceeded to experimental data. The analytical model data was replaced by the experimental data, and the network was retrained. In this case, we considered the possibility that one may not be able to measure a very dense admittance spectrum, and so we resampled the spectra for a variable number of points $N_p$ between 100 and 1601. We present the accuracy for the test data set as a function of $N_p$ in Fig. 10. Each data point was trained using $10^4$ iterations. Below 201 points, the accuracy drops significantly, while above this value, there is a steady and slow increase in accuracy. Using the full spectrum of 1601 points, we achieve over $98\%$ localisation accuracy for all objects with a size comparable with that of the helical resonator’s period (11 mm). Since the size of the neural network required for localisation is directly related to the number of spectrum points used, being able to reduce this number should both simplify and speed up the processing for localisation. This supports the idea that smaller architectures which can be embedded into relatively low memory chips can be achieved. To compare the actual size of the model, an architecture with 1601 features results in a 657 kB model, which drops to 337 kB for 801 features, 177 kB for 401 features and to 97 kB for 201 features. The reduction in model dimension results in lower accuracy, which can be partly mediated by further training.

**Fig. 11:**
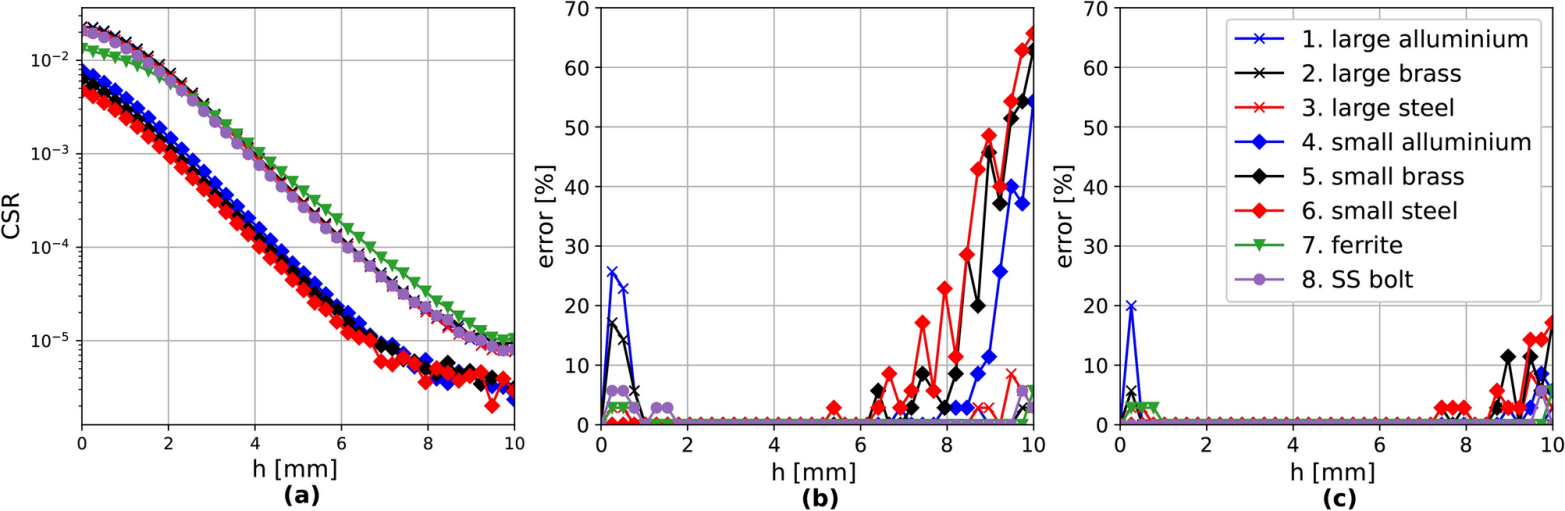
Experimental localisation results with 1601 features: (**a**) mean change to signal ratio as a function of altitude, *h*, (**b**) accuracy for all objects as a function of altitude, *h*, before retraining and (**c**) after retraining.

An additional noteworthy feature is the poorer relative performance for smaller objects when compared with all the other objects. This is expected as the magnitude of the change they generated in the meta-atoms of the surface is significantly smaller. This is illustrated by the results in Fig. 11a, which displays the previously defined mean change to signal ratio (CSR) as a function of vertical location, *h*. CSR is computed using the maximum object altitude signal at location ${\textbf {1a}}$ as the baseline. This is to ensure that the algorithm does not model changes in the background caused by cable positions and environment variations. The magnitude of change associated with the small objects is more than half an order of magnitude lower. This explains the lower accuracy of the small objects - the model did not manage to fit the small changes after $10^4$ iterations.

Figure 11b displays the percentage of mislabelled locations for each object altitude when 1601 $|\Delta S_{11}|$ spectrum points are used. There are two significant features of this graph. The first is the deterioration of the small objects’ accuracy at altitudes above $h=6$ mm, which we have already associated with the small CSR of these objects. To mediate this issue, the small objects were further trained for a total of $5\times 10^4$ iterations. Considering that the size of the objects is only half of the period of a meta-atom, the need for further retraining should be expected as a consequence of the smaller $|\Delta {S_{11}}|$ changes these objects will cause. The second noteworthy feature is the low accuracy for the large aluminium, large brass, and SS-bolt objects at altitudes between 0 and 1 mm. This is caused by the strong discontinuity in the quality factor trend that occurs for altitudes below 0.5 mm, as shown in Fig. 5. We believe that this arises thanks to extra electrostatic effects, whose range is extremely short. To somewhat mediate for this, these three objects were retrained with a new training data set where the data at altitude $h =0.5$ mm was also included.

After mediating these two issues by increasing the number of training iterations for the small objects (small aluminium, small brass and small steel) and adding extra training data for the large aluminium, large brass and SS-bolt, the errors in localisation have significantly improved. The error for each altitude layer is shown in Fig. 11c, where nearly error-free (<2%) localisation is now possible for altitudes between 1 and 8 mm for all objects. Table 2 shows the accuracy for the full data set (training and testing combined) before retraining and after retraining.

**Table 2:** Accuracies for all objects on the full data set before and after retraining.

Object id	Object	Accuracy before re-training (%)	Accuracy after re-training (%)
1	Large aluminium	98.5	99.4
2	Large brass	98.7	99.7
3	Large steel	99.2	99.2
4	Small aluminium	94.5	99.5
5	Small brass	90.0	98.7
6	Small steel	88.0	98.2
7	Ferrite	99.5	99.5
8	SS-bolt	98.9	99.7

### Separation results

**Fig. 12:**
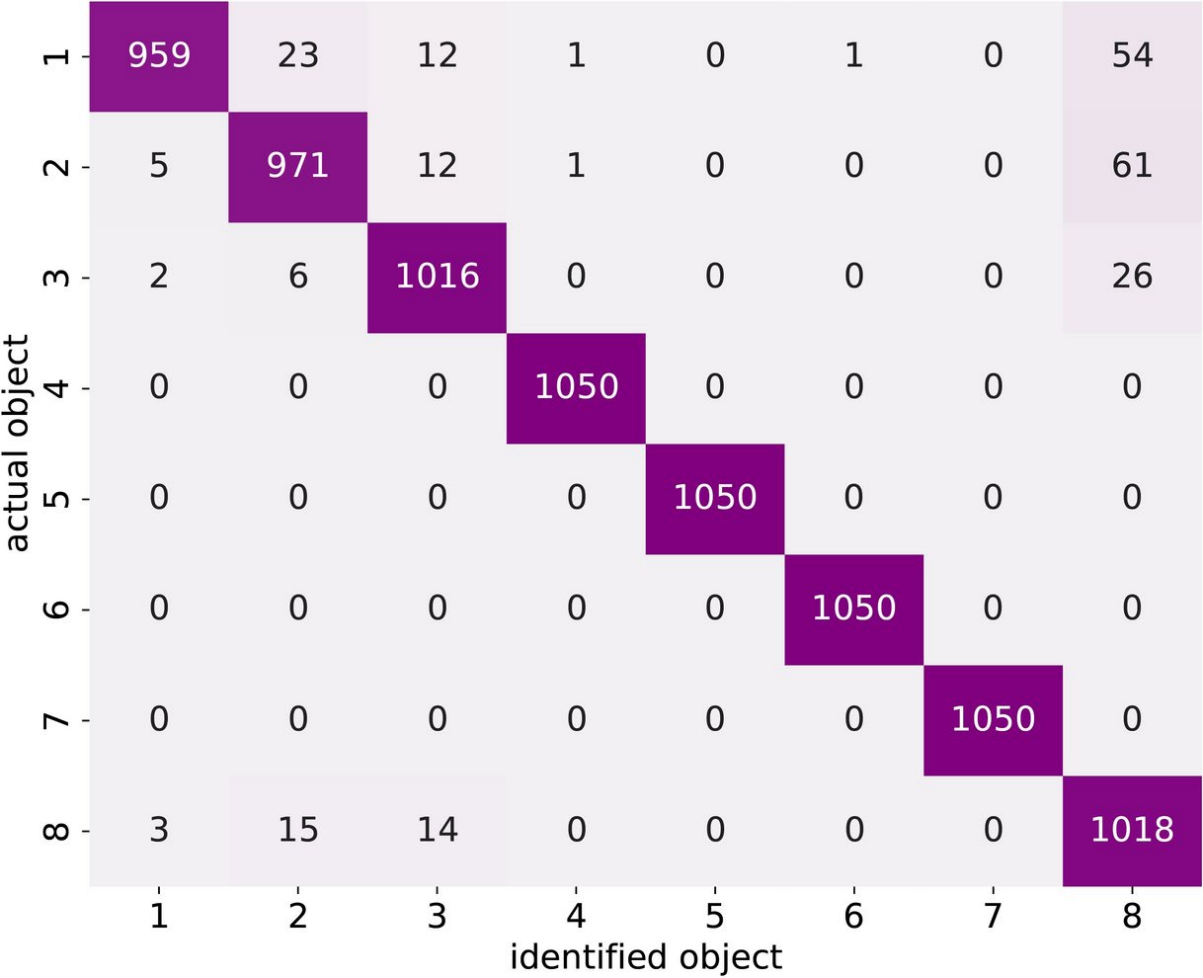
Confusion matrix for the test data set showing the number of times each object was identified correctly (diagonal cells) and misidentified as each of the other objects (off-diagonal cells).

The same principle was involved in classifying objects. A number of $10^5$ iterations was used to train the model. As in the case of CSR estimation, it was decided to use a high object altitude data for normalisation. This is very important as we want to ensure we don’t train on the differences that have accumulated between different measurement runs.

To show our classification performance, where we ask the network to identify which object has been presented to the metasurface, we show the ’Confusion matrix’ in Fig. 12. This shows the number of times a certain object (labelled as *actual object* in Fig. 12) has been identified as any of the other objects (labelled as *identified object* in Fig. 12). The overall accuracy of the algorithm for the testing dataset is $97.2\%$, and for the full dataset is $97.9\%$.

### Cross training results

In a real applications of our sensing platform, storing a separate model for every possible object would be impossible, however creating a model for different classes of objects is quite achievable. In this section, the possibility of grouping objects by material type and / or size is explored.

**Fig. 13:**
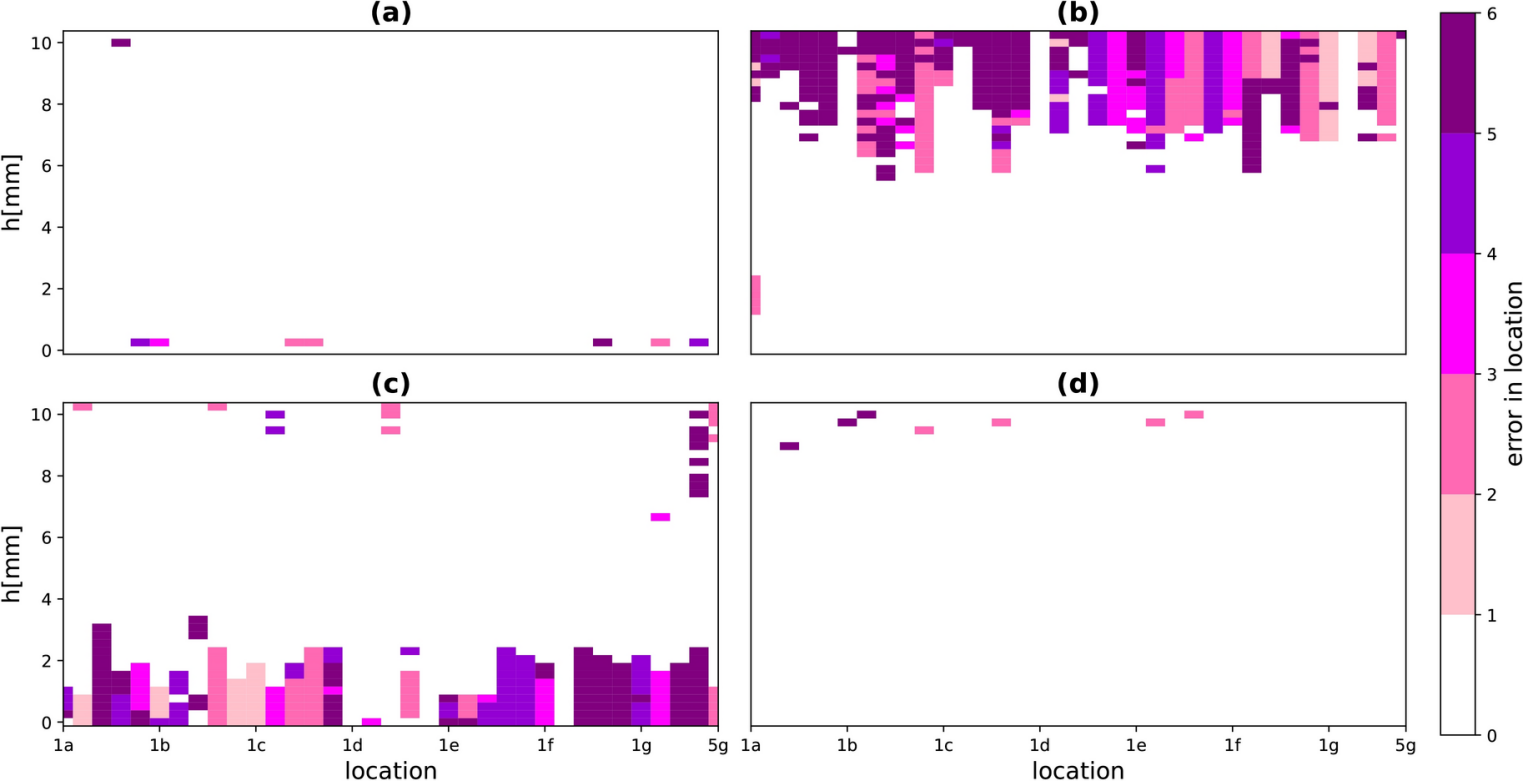
Cross training error in localisation between large and small aluminium discs: (**a**) training with the large object and testing with the large one, (**b**) training with the large object and testing with the small one, (**c**) training with the small object and testing with the large one and (**d**) training with the small object and testing with the small one. The error in localisation is computed as the Manhattan distance between the correct location and the estimated one.

Starting with the separation by object material, Fig. 13 shows the difference in estimated position and actual position as a function of altitude (*h*) and location when the two aluminium disc objects (large and small) are used in both possible combinations of training and testing. With these two sets of data we can use one to provide the training subset as usual but then having trained the system on that object’s data we can present the data from the other object and hence determine if estimated location is still good. Training and testing on the same objects already give good results (Fig. 13a and d). When using the model trained on the small aluminium disc and testing on the large aluminium objects, the localisation accuracy is poor for low altitudes below $h = 2.5$ mm. Conversely, training with the large disc dataset and testing with the small one gives low accuracy for altitudes above $h=6$ mm. These results, far from being discouraging, are actually very useful. Looking back at the data evaluating the objects’ interaction with a meta-atom presented in Fig. 5, it’s possible to see what is happening. The range of resonant frequencies covered by the small objects is a subset of those generated by the large objects. For example the large aluminium disc detunes the meta-atom from $183 \ \textrm{MHz}< f_{\textrm{res}} < 226 \ \textrm{MHz}$ ($130> Q > 80$) whilst the small aluminium disc only covers a range from $183 \ \textrm{MHz}< f_{\textrm{res}} < 190 \ \textrm{MHz}$ ($130>Q > 118$) for the same altitude range. This means there are data whose $|\Delta S_{11}|$ values cover the ’small’ object range within the ’large’ object set. In other words, the large objects at greater altitudes are indistinguishable from the small objects in closer proximity to the metasurface, which is shown neatly in Fig. 13b. Equally, there are $|\Delta S_{11}|$ values in the ’large’ set with no equivalent in the ’small’ training data. This means that the stronger signals generated by the ’large’ object will be misinterpreted by a network trained on the ’small’ dataset. Conversely, when training with large data, there are too few altitudes for which the CSR covers the small values, resulting in inaccuracies from limited training data.

One could, therefore, create a new categorisation of the objects based on their material. The objects will be as follows: (1) aluminium objects, (2) brass objects, (3) steel objects, (4) ferrite and (5) SS bolt. If the same procedure as before is applied for localisation and each model is trained with $5\times 10^4$ iterations, the full data set accuracy for the newly created categories are: (1) $98.0\%$ (2) $98.3\%$ and (3) $98.6\%$ with the accuracy for the SS bolt and ferrite remaining the same as before. Additionally, separation (i.e. determining the nature of the object being presented) between these five categories is possible with accuracy $96.7\%$ for the test dataset and $97.5\%$ for the full dataset after $5\times 10^4$ iterations.

The same principle can be applied to separate objects by size. One can create three new categories: (1) large objects (containing SS bolt, large aluminium, large brass and large steel), (2) small objects (containing small aluminium, small brass and small steel) and (3) ferrite. The accuracy of localisation for the two new categories on the full data set are (1) $98.9\%$ and (2) $99.5\%$. As before, the large objects were trained for $10^4$ iterations and the small objects for $5\times 10^4$ iterations. The same training to testing split of 3:1 was used for both objects with no additional layer added for the large metal object. The separation accuracy between these three categories for the test data set is $99.5\%$, and for the full data set is $99.6\%$. In other terms, this implies that our metasurface can unambiguously differentiate between our objects’ composition and size, then use the appropriate network to achieve localisation to better than $99\%$ accuracy.

## Conclusion and discussion

In this paper, we have reported a novel method for implementing the two-dimensional localisation of objects using a metasurface. Our method exploits the spatial distribution of standing waves and its modification by the impact of an object to encode position information onto the frequency response of the metasurface. Whilst this is similar to the capabilities of a capacitive touchscreen, here we utilise a single electrical feed point to the entire meta-surface in order to locate objects on it. By analysing the input admittance spectrum of that feed, we are also able to identify objects distinguishing between them on a $7\times 5$ metamaterial array operating at 183 MHz. Our method exploits both the properties of waves in the metasurface and a machine-learning approach.

A simple, three-layer neural network was chosen to model the complex behaviour of a metasurface due to its high accuracy, ease of implementation, and low inference time once the network was trained. A number of 365 samples were used for training, which was sufficient for our system where the signal-to-noise ratio is close to 80 dB and additional, repeated measurements are therefore unnecessary. This is supported by the accuracy of the network, which exceeds $98\%$ on the testing datasets. The system presented is a proof-of-concept, and further improvements for larger or more complex applications can be obtained by employing a more complicated neural network architecture and collecting more samples for the same location and altitude. From a practical perspective, a larger dataset would result in a network capable of addressing deviations and noise in more challenging environments than the one presented in this work.

From an implementation perspective, an important point of discussion was the position of the feeding/measurement point. It was shown that an asymmetrical feed can ensure that each location results in a unique change in the input impedance spectra when an object is placed immediately above it. The results obtained were in line with the expectations: a central feed with only 12 unique object locations has an accuracy slightly below 12/35, a side feed with 21 unique object locations has a greater accuracy slightly below 21/35, and an asymmetric feed with 35 unique object locations has an accuracy close to 1. This supports the fact that localisation on a metasurface is only possible with an appropriate choice of feeding location. We note that the introduction of disorder to the metasurface could also play a role in the future improvement of this result.

Furthermore, experimentally, different objects were located with accuracies above $98\%$ up to a distance above the surface of 10 mm. This shows that a neural network is an effective way of modelling a complex system for defects of different conductivity, size and shape. The size of the objects and the altitudes of the objects are strongly correlated in terms of impact on detection. A smaller object would generate smaller changes to meta-atom equivalent circuit parameters, hence being more difficult to see at higher altitudes. A smaller object thus requires finer tuning of the neural network coefficients in order to account for the smaller changes that occur. As a result, for the smaller diameter objects, we have increased the number of training iterations by a factor of five and modified the learning rate such that it goes down by $10\%$ for every 10000 iterations. This resulted in a detection accuracy close to $100\%$ up to 8 mm. At higher altitudes, the small samples’ performance begins to deteriorate, indicating that for the altitude range considered, the small samples, whose diameter is approximately half of the coil period, are close to the limit of detection that can be achieved. For the upper limit on the object size, one must be able to distinguish between the effect on two meta-atoms. If the object is larger than six times the lattice period, we expect that many of the meta-atoms in the middle of the object will be similarly impacted, as we have found in^35^, and resolving between the two resonators will be difficult.

Different objects (material and size) were also found to have differing interactions with the surface and in consequence, their identification was possible with an accuracy above $97\%$ up to a distance of 10 mm.

Considerations for a practical system were also studied. The number of features in the admittance spectrum was varied to assess the ability to use this technique with VNAs capable of recording fewer points, as well as the ability to have smaller models that can be embedded in commercially available microcontroller chips. It was found that with 201 spectral points, one can expect an accuracy of roughly $85\%$, which increases to more than $90\%$ for 401 features. Careful selection of these features by identifying the peaks and troughs of the spectrum is an area of further research that can significantly speed up the process and hardware requirement for the model.

Another practical consideration was the impact of noise. It was found that for normal VNAs at 183 MHz, accuracy is expected to remain above $86\%$ in the worst-case scenario. This can also be mitigated through the use of filtering and smoothing techniques during the post-processing step.

A final analysis was made on cross-training capabilities to assess whether or not detecting an unknown object similar to one already stored could be achieved. It was found that this was possible in a limited altitude range as long as the object’s impact on the surface is comparable to the set standard in terms of resonant frequency and quality factor. It was also found that grouping objects is possible to simplify the number of models required and to generalise the type of objects under test.

Overall, our metasurface localiser has demonstrated a robust capability similar to that of a touchscreen, albeit at lower spatial resolution, whilst only requiring a single electrical feed point to function. These metasurfaces are scaleable, meaning that the same system will work even on large room-scale structures like floor coverings. As well as detecting an object in contact or proximity, our metasurface can also be designed to detect damage to its meta-atoms, which is very similar to the effect of a large metal object placed in contact with them. Hence, we anticipate applications for this technology in surfaces where damage detection is important, such as vehicles, clothing and buildings. This work has supported the use of machine learning techniques for modelling known complex phenomena and went further by enabling the extraction of spatial information from spectral data.

## Data Availability

Experimental data and codes are available by request from the corresponding author.
